# Genomic and Metabolomic Analyses of *Streptomyces albulus* with Enhanced ε-Poly-l-lysine Production Through Adaptive Laboratory Evolution

**DOI:** 10.3390/microorganisms13010149

**Published:** 2025-01-13

**Authors:** Xidong Ren, Xinjie Sun, Yan Chen, Xiangheng Xi, Yunzhe Ma, Xinyue Jiang, Xian Zhang, Chenying Wang, Deqiang Zhu, Xinli Liu

**Affiliations:** 1 State Key Laboratory of Biobased Material and Green Papermaking, Qilu University of Technology, Shandong Academy of Sciences, Jinan 250353, China; renxidong1986@126.com (X.R.); zdq0819@qlu.edu.cn (D.Z.); vip.lxl@163.com (X.L.); 2 Shandong Provincial Key Laboratory of Microbial Engineering, Department of Bioengineering, Qilu University of Technology, Shandong Academy of Sciences, Jinan 250353, China; 3 Key Laboratory of Industrial Biotechnology of Ministry of Education, School of Biotechnology, Jiangnan University, Wuxi 214122, China; 4 School of Food Science and Engineering, Qilu University of Technology, Shandong Academy of Sciences, Jinan 250353, China

**Keywords:** *Streptomyces albulus*, ε-poly-l-lysine, adaptive laboratory evolution, genomics, metabolomics

## Abstract

ε-poly-l-lysine (ε-PL), a natural food preservative, has garnered widespread attention. It is mainly produced by *Streptomyces albulus*, but the production by wild-type strains fails to meet the demands of industrialization. To address this issue, adaptive laboratory evolution (ALE) was successfully employed in this study, subjecting *S. albulus* CICC 11022 to environmental stresses such as acidic pH and antibiotics (rifampicin, gentamicin, and streptomycin). As a result of ALE, an evolutionary strain *S. albulus* C214 was obtained, exhibiting an increase in ε-PL production and cell growth by 153.23% and 234.51%, respectively, as compared with the original strain. Genomic and metabolic analyses revealed that mutations occurred in genes responsible for transcriptional regulation, transporter, cell envelope, energy metabolism, and secondary metabolite synthesis, as well as the enrichment of metabolites involved in the biosynthesis of ε-PL. These findings hold great significance for elucidating the mechanism underlying ε-PL synthesis.

## 1. Introduction

ε-Poly-l-lysine (ε-PL) is a non-ribosomal polypeptide primarily biosynthesized by *Streptomyces albulus*, consisting of 25–35 lysine residues [1]. The ε-PL has broad antimicrobial spectra against various Gram-positive and Gram-negative bacteria, fungi, yeasts, and specific viruses. The previous study demonstrates that the antimicrobial mechanism of ε-PL predominantly involves disrupting cell membrane integrity and inducting oxidative stress through reactive oxygen species (ROS) generation [2]. The high safety and remarkable characteristics of ε-PL make it a promising preservative in the food industry, including fruits, beverages, fish, salad dressings, and cheese [3]. Additionally, ε-PL has been used in bio-based wood preservation [4], antimicrobial therapeutics [5], fibrous membrane wound dressings [6], and nanotechnology [7].

Generally, ε-PL production by wild-type strains is limited, while strain improvement would greatly enhance ε-PL production. The breeding methods commonly employed in current research include random mutagenesis, genome shuffling, ribosome engineering, and genetic engineering, either individually or in combination [8]. The production of ε-PL was enhanced through atmospheric and room temperature plasma (ARTP) mutagenesis, resulting in an 18.46% increase in ε-PL production [9]. Six antibiotics were successively introduced to the cultivation of *S. albulus* FEEL-1, resulting in a multi-antibiotic-resistance strain, *S. albulus* R6, which exhibited a 2.75-fold increase in ε-PL production [10]. Liu et al. [3] employed genome shuffling to obtain a higher ε-PL-producing strain, *S. albulus* SG-86, which showed a 144.7% increase in ε-PL production. The heterologous expression of the AraC family transcription factor (*AdpA*) has effectively promoted both ε-PL production and sporulation in *S. albulus* NK660, leading to an approximate 3.6-fold enhancement in ε-PL production [11]. Yang et al. [12] engineered a polyphosphate kinase (PPK)-mediated ATP regeneration system in *S. albulus* WG608, and ε-PL production reached 2.34 g/L in shake-flask fermentation, with an increase of 21.24% when compared with the original strain.

In recent years, adaptive laboratory evolution (ALE) has gained significant popularity in strain improvement. ALE involves continuous cultivation of the target strain under survival stresses, leading to numerous non-directed beneficial mutations in the microorganism’s genome. These mutations result in the simultaneous activation of multiple stress response mechanisms within the cell, thereby enhancing both microbial survival capabilities and metabolite production [13]. For instance, through seven different competition experiments involving three serial passages, the *Streptomyces* strain JB140 developed seven unique mutant phenotypes that exhibited enhanced antimicrobial activity against bacterial pathogens. These mutant phenotypes not only effectively inhibited the growth of the tested pathogens, but exhibited improved antimicrobial responses against a clinical multidrug-resistant (MDR) uropathogenic *Escherichia coli* (UPEC 1021) isolate [14]. Charusanti et al. [15] demonstrated that when multiple replicates of *S. clavuligerus* adaptively evolved against methicillin-resistant *Staphylococcus aureus* N315, a strain emerged with the ability to constitutively produce holomycin. In contrast, no holomycin production was detected in the unevolved wild-type strain. These findings suggest that ALE can serve as a powerful tool for discovering and enhancing antibacterial-producing strains.

In the present study, an ε-PL-producing strain *S. albulus* CICC 11022 was subjected to multiple antibiotics (rifampicin, gentamicin, and streptomycin) as well as acidic pH stresses in ALE. Consequently, an evolved strain with enhanced ε-PL production was selected. Subsequently, a comparative analysis of fermentation performance, genome alterations, and metabolome changes was conducted between the original and evolved strains to elucidate the underlying mechanisms responsible for the enhancement of ε-PL production.

## 2. Materials and Methods

### 2.1. Strain, Media, and Seed Culture Conditions

*S. albulus* CICC 11022 was acquired from the China Center of Industrial Culture Collection (CICC). The BTN medium used for solid culture contained (g/L) glucose 10, yeast extract 1, tryptone 2, and agar 20, with pH 7.0. The M3G medium used for seed culture, fermentation (pH 6.8), and ALE (pH 4.0), was composed of glucose 50, yeast extract 5, (NH_4_)_2_SO_4_ 10, MgSO_4_·7H_2_O 0.5, ZnSO_4_·7H_2_O 0.03, FeSO_4_·7H_2_O 0.03, KH_2_PO_4_·2H_2_O 1.4, and K_2_HPO_4_·2H_2_O 0.8. The slants were inoculated and incubated at 30 °C for 7 days to obtain a heavy sporulated growth. A loop of spores was inoculated into 50 mL M3G medium in a 300 mL shake flask and incubated on a rotary shaker at 200 rpm and 30 °C for 24 h.

### 2.2. Mutagenesis by ALE

Acidic pH and multiple antibiotics were employed as environmental stresses for ALE. *S. albulus* CICC 11022 was cultured at an initial pH of 4.0, while rifampicin, gentamicin, and streptomycin were sequentially utilized. Prior to subjecting *S. albulus* to antibiotic-induced environmental stress, it is essential to determine the minimum inhibitory concentration (MIC) of the initial strain. To achieve this, 100 μL spore suspensions of *S. albulus* were inoculated onto BTN plates containing varying concentrations of antibiotics. The MIC was defined as the lowest antibiotic concentration that inhibited visible growth, resulting in minimal colony formation on the plates. The MIC values of the antibiotics were determined as follows: rifampicin (0.4 mg/L), gentamicin (18 mg/L), and streptomycin (190 mg/L). The seed cultures (2 mL) were transferred to fresh M3G medium (pH 4.0, 50 mL) containing antibiotics (1 MIC), and incubated at 200 rpm and 30 °C for 4 days, during which, the pH spontaneously dropped to approximately 3.0 after the first day. The antibiotic concentration was elevated by 1 MIC or changed to another antibiotic after each round of transference and cultivation until ε-PL production ceased to increase further. At regular time intervals, the culture was diluted and spread on BTN plates (pH 4.0) using the same antibiotic concentration as in ALE. Single colonies exhibiting high resistance against both antibiotic and acid stresses were selected for evaluating ε-PL production through shake flask fermentation.

### 2.3. Batch Fermentation

Two milliliters of seed cultures were inoculated into the 50 mL M3G medium (pH 6.8) in a 300 mL shake flask and cultured at 200 rpm and 30 °C for 72 h. Samples were collected at intervals of 6 h to measure pH, dry cell weights (DCW), and ε-PL production.

### 2.4. Whole-Genome Resequencing

A loop of *S. albulus* C214 spores was inoculated into M3G medium and cultured at 200 rpm and 30 °C for 20 h. Following two washes with PBS solutions, DNA extraction was performed. The quantity and integrity of DNA were separately assessed using a fluorescent dye (Quant-iT PicoGreen dsDNA Assay Kit, Thermo Fisher Scientific, Waltham, MA, USA) and 1% agarose gel electrophoresis. The Illumina TruSeq Nano DNA LT library protocol (Illumina TruSeq DNA Sample Preparation Guide, Illumina, SD, USA) was used to construct the genomic library. Then, high-throughput sequencing was performed. The original offline data (raw data) were filtered to generate high-quality sequences (high-quality data). The raw data of high-throughput sequencing for *S. albulus* C214 were deposited in the NCBI database under BioProject accession number PRJNA1204205. The bwa (0.7.17-r1188) mem program was used to align the high-quality data to the *S. albulus* CICC 11022 reference genome (GenBank no. ASM385166v1). To obtain all mutation points of the sample, the UnifiedGenotyper program tool from the Genome Analysis TK v3.8 software package was employed, with stand_call_conf set at 30 and stand_emit_conf set at 10 for SNP loci identification, and both stand_call_conf and stand_emit_conf set at 10 for the identification of InDel mutations. Finally, SNP and Indel sites were annotated using ANNOVAR software.

### 2.5. Metabolite Analysis

The above cultures were washed twice with PBS solutions and centrifuged at 5000× *g* for 10 min. The resulting precipitates were mixed with a methanol solution (50%, *v*/*v*), rapidly frozen in liquid nitrogen for 5 min, and ground at 55 Hz for 2 min. After centrifugation at 4 °C (12,000× *g*, 10 min), the supernatant was first freeze-dried and then redissolved in a 300 μL solution of 2-Amino-3-(2-chloro-phenyl)-propionic acid (4 ppm) prepared with a methanol solution (50%, *v*/*v*). The redissolved supernatant was filtered through a 0.22 μm membrane and transferred for LC-MS (Vanquish UHPLC System-Q Exactive, Thermo Fisher Scientific, Waltham, MA, USA) detection. The raw data were first converted to mzXML format by MSConvert using the ProteoWizard software package (v3.0.8789) and processed using XCMS (v3.10.1) for feature detection, retention time correction, and alignment. Metabolites were identified based on accurate mass measurements (<30 ppm) and MS/MS data that matched entries in HMDB, MassBank, LipidMaps, mzCloud, and KEGG. Multivariate data analyses and modeling were performed using ropls software (v3.1.2). Models were built based on principal component analysis (PCA), orthogonal partial least-square discriminant analysis (PLS-DA), and partial least-square discriminant analysis (OPLS-DA). Metabolites with *p* value  < 0.05 and VIP > 1 were considered statistically significant.

### 2.6. Measurement of ε-PL Production and Dry Cell Weights

The fermentation broth was centrifuged at 5000× *g* for 10 min to obtain a supernatant for ε-PL production determination [16]. The resulting precipitate was filtered using pre-weighed filter paper, dried at 105 °C to a constant weight, and then used to measure the dry cell weights (DCW).

### 2.7. Statistical Analysis

To ensure reproducibility, the experiments were conducted in triplicate or more. Statistical significance was assessed using SPSS Statistics 20 (IBM, Armonk, NY, USA) through a one-way analysis of variance (ANOVA), followed by Tukey’s honestly significant difference (HSD) post hoc test (*p* ≤ 0.05).

## 3. Results and Discussion

### 3.1. Screening of Evolved S. albulus with Higher ε-PL Production by ALE

**Figure 1:**
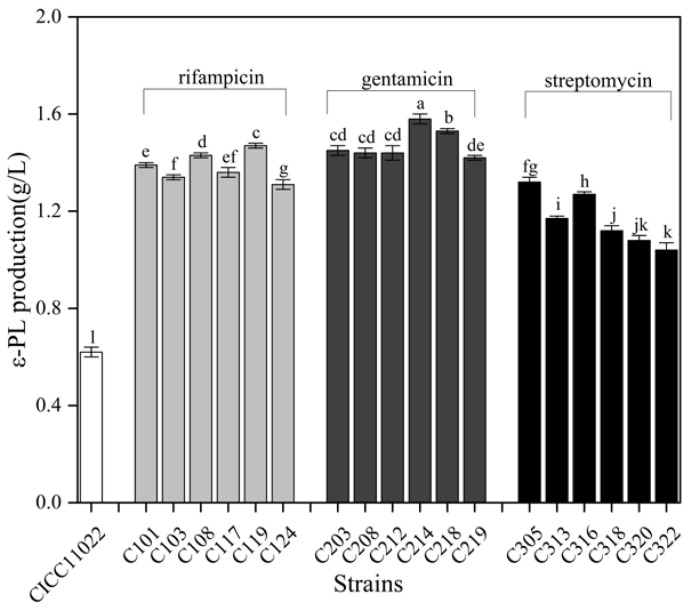
ε-PL production by mutants screened from the ALE of acidic pH and antibiotics. Statistical significance is denoted by different letters (*p* ≤ 0.05).

It has been reported that the antibiotic-resistant mutants of *Streptomyces* could enhance the production of secondary metabolites [17]. As shown in Figure 1, *S. albulus* C119 exhibited the highest ε-PL production after the ALE of rifampicin stress, which was 137.10% higher than that of the *S. albulus* 11022 and was selected as the starting strain for the subsequent ALE of gentamicin stress. After the ALE of gentamicin, the highest ε-PL production was obtained by *S. albulus* C214, which increased by 7.48% compared with that of *S. albulus* C119. When *S. albulus* C214 was finally subjected to the ALE of streptomycin stress, the ε-PL production values of the obtained ALE derivatives all decreased. The effectiveness of antibiotic stress on improving ε-PL production decreased as the duration of evolution increased. Likewise, Liu et al. [17] found that ε-PL production by resistant mutants, screened from 6 μg/mL streptomycin cultures, increased from 1.60 to 2.59 g/L, while ε-PL production reached 3.04  g/L when the streptomycin concentration was raised to 30 μg/mL before decreasing again to 2.55 g/L when streptomycin was finally raised to 150 μg/mL. The mutations associated with antibiotic resistance mainly affected ribosomal protein S12, RNA polymerase, and other ribosomal proteins. The translation factors, such as the *rpsL* gene encoding ribosomal protein S12, were found to be mutated in streptomycin-resistant mutants, while the *rpoB* gene encoding the RNA polymerase β-subunit protein was found to be mutated in rifamycin-resistant mutants [18]. Therefore, the promotion of secondary metabolites would gradually decrease with the extension of antibiotic-induced ALE. This observation suggested that short-term antibiotic ALE is more suited for activating secondary metabolites.

### 3.2. Comparisons of ε-PL Production by S. albulus CICC 11022 and S. albulus C214 in Batch Fermentation

**Figure 2:**
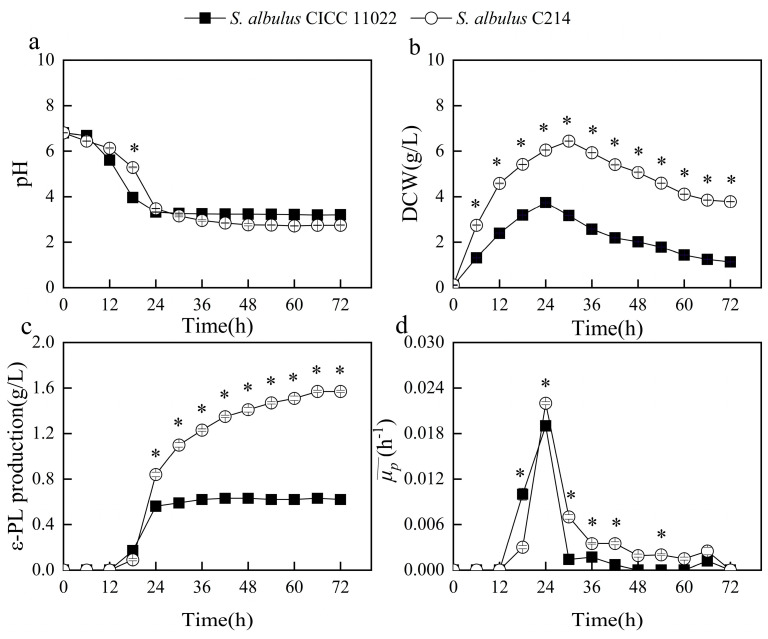
Time profiles of (**a**) pH, (**b**) DCW, (**c**) ε-PL production, and (**d**) $\bar{\mu_{p}}$ by the original and evolved strains during batch fermentation. Statistical significance is denoted by * at each time point (*p* ≤ 0.05).

To evaluate the fermentation performances, comparisons of pH, DCW, ε-PL production, and the average specific ε-PL formation rate ($\bar{\mu_{p}}$) were made in shake-flask fermentation between the original strain of *S. albulus* CICC 11022 and the evolved high-producing strain of *S. albulus* C214. The pH values of both strains initially decreased and subsequently stabilized, in accordance with our previous study [16]. However, it is worth noting that while the pH of *S. albulus* CICC 11022 decreased to approximately 3.2, that of *S. albulus* C214 dropped to about 2.7 (Figure 2a). Notably, cell growth and ε-PL production were significantly enhanced in the mutant when compared with the original strain. The DCW and ε-PL production values of *S. albulus* C214 reached their maximum at 3.78 and 1.57 g/L, which were 234.51% and 153.23% higher than those of *S. albulus* CICC 11022 (Figure 2b,c). It should be noted that when the environmental pH fell below 3.2, both strains experienced extreme acid stress, leading to reduced DCW as well as slowed or even ceased ε-PL production [19]. The $\bar{\mu_{p}}$ values of both strains initially increased, then decreased, and eventually stabilized (Figure 2d). Before 18 h, the $\bar{\mu_{p}}$ of *S. albulus* CICC 11022 was higher than that of *S. albulus* C214. This can be attributed to the fact that the pH of *S. albulus* C214 (about 5.5 at 18 h) was not conducive for ε-PL production, whereas the pH of *S. albulus* CICC 11022 had already decreased to a suitable level (about 4.0 at 18 h) for ε-PL production. However, after 18 h, the $\bar{\mu_{p}}$ of *S. albulus* C214 consistently exceeded that of *S. albulus* CICC 11022, indicating that the ε-PL production capability of individual cells was also enhanced after ALE. Among these effects observed under acid stress conditions, it was evident that the impact of acid stress on the original strain was significantly greater than that on the evolved strain, which means that the mutant obtained higher acid resistance after ALE.

### 3.3. Genomic Analysis

To investigate the genomic changes after ALE, whole-genome resequencing was carried out. The results revealed that the mutant *S. albulus* C214 exhibited a total of 7 single nucleotide polymorphisms (SNPs) and 118 insertion–deletions (InDels) when compared with the original strain *S. albulus* CICC 11022. The GO and KEGG annotations revealed that the mutant genes were primarily associated with transcriptional regulation, transporter, cell envelope, energy metabolism, and secondary metabolite synthesis (Table 1).

#### 3.3.1. Transcriptional Regulation

The RNA polymerase sigma-70 factor, ECF subfamily (σ^E^), encoded by SALB_RS22375, plays a pivotal role in maintaining cell wall homeostasis, responding to oxidative and surface stresses, and modulating the transcription of respective response genes [20]. The genes SALB_RS03255, SALB_RS15530, and SALB_RS11990 encode transcriptional regulators belonging to the XRE, MerR, and LysR families, respectively. The XRE family transcriptional regulator could autoregulate its own transcription. In the absence of this regulator, the development of aerial mycelia and spores in *S. coelicolor* was inhibited while antibiotic production was enhanced [21]. It has been demonstrated that the MerR family transcriptional regulator can enhance the affinity of promoters [22]. Furthermore, both MerR and LysR family transcriptional regulators are capable of conferring antibiotic resistance to the strains [23,24].

#### 3.3.2. Transporter

The robust transportation systems of *Streptomyces* enable it to effectively adapt to intricate environments. The ABC transporters encoded by SALB_RS20610 and SALB_RS11770 are classified as primary active transporters, primarily responsible for nutrient uptake and intracellular substance secretion (mainly secondary metabolites) [19]. Hillerich and Westpheling [25] demonstrated the involvement of ABC transporter Agl3EFG in carbohydrate transport triggered by glucoside signaling, thereby influencing mycelial differentiation and antibiotic production in *S. coelicolor*. The MFS transporter belongs to the category of electrochemical potential-driven transporters. In this study, six genes (SALB_RS21360, SALB_RS43140, SALB_RS42470, SALB_RS43175, SALB_RS21805, and SALB_RS43140) annotated as MFS transporters were found to be mutated. These transporters are primarily responsible for the transportation of sugars and organic acids while playing a significant role in drug efflux [26]. For instance, Nag et al. [27] demonstrated that SCO4121, an MFS transporter from *S. coelicolor,* is involved in multidrug resistance and oxidative stress tolerance. In response to the acidic pH and antibiotic stresses in ALE, *S. albulus* CICC 11022 transporters underwent a series of mutations that regulated the nutrient uptake and intracellular metabolite efflux to ensure intracellular homeostasis. A membrane ATPase (*atp*) encoded by SALB_RS31895 could function as a proton pump by hydrolyzing ATP to expel intracellular H^+^, thus alleviating intracellular acidification in ALE.

#### 3.3.3. Cell Envelope

The lyso-ornithine lipid O-acyltransferase (encoded by SALB_RS17270, *olsA*) was mutated in *S. albulus* C214, affecting the biosynthesis of ornithine lipids. Stress conditions can lead to reduced phospholipid production, while ornithine lipids can serve as an alternative to phospholipids under phosphate-limiting conditions [28]. Given that the ornithine moiety of ornithine lipids is exposed to the extracellular environment, the bacterial surface properties are expected to change when ornithine lipids substantially replace phospholipids. Because the membrane function serves as a protective barrier of cells against harmful external environments, alterations in membrane composition can enhance cellular resistance to external acid and antibiotics [29]. The mutated [acyl-carrier-protein] S-malonyltransferase (encoded by SALB_RS01500) and fatty acid synthase (encoded by SALB_RS43310, *FASN*) were both involved in the biosynthesis of membrane fatty acids. The SALB_RS28520 gene-encoding cholesterol oxidase was mutated in *S. albulus* C214. Cholesterol, a crucial component of the cellular membrane found universally across organisms, plays an essential role in maintaining membrane fluidity and normal physiological functions. It also contributes to lipid membrane flexibility and enhances the resistance of liposome vesicles against external environmental changes. Moreover, the CDP-glycerol glycerophosphotransferase (encoded by SALB_RS44660) facilitates the production of wall teichoic acid (WTA), which is subsequently linked to the biosynthesis of peptidoglycan in the cell wall [30]. Sigle et al. [31] demonstrated that WTA contributes to thickening the spore envelope in *Streptomyces*, enhancing their ability to withstand adverse external conditions. It has been deduced that the mutation in this gene could confer a protective effect on the cell wall against the detrimental impact of acidic pH and antibiotics in ALE. In our previous study, we also identified MreB as the rod shape-determining protein capable of thickening the spore envelope, which contributes to acid tolerance in *S. albulus* [19].

#### 3.3.4. Energy Metabolism

In terms of energy metabolism, mutations were introduced in NAD(P)H dehydrogenase (encoded by SALB_RS44690, *NQO1*), AMP-dependent synthase (encoded by SALB_RS44480), and FAD-binding oxidoreductase (encoded by SALB_RS37945). The NAD(P)H dehydrogenase plays a role in initiating and maintaining electron transfer, which provides the power source for ATP synthesis. The AMP-dependent synthase plays a pivotal role in regulating cellular energy metabolism. When ATP levels are depleted, the enzyme is activated in response to an increase in AMP concentration, thereby stimulating ATP production to meet the demands of energy metabolism [32]. FAD-binding oxidoreductase facilitates the reactions of FAD-NADH oxidase and NADH-FDA reductase, resulting in energy generation and reduction of ROS to H_2_O. Consequently, it actively participates in cellular oxidative stress response and energy metabolism while effectively scavenging excessive intracellular ROS, attenuating cellular damage while providing essential energy for metabolic processes [33]. During ε-PL production by *Streptomyces*, cell membrane disruption and ROS accumulation often emerged as primary factors leading to bacterial mortality [34], whereas intracellular ATP accumulation can benefit both ε-PL production and acid tolerance [16]. Therefore, mutations in genes involved in redox reactions and energy metabolism might enhance cellular viability and productive performance of ε-PL production.

#### 3.3.5. Secondary Metabolite Synthesis

The synthesis of secondary metabolites involves a complex interplay of diverse enzymes, among which polyketide synthases (PKS) form a prominent and extensive family. In this study, five genes (SALB_RS42445, SALB_RS31335, SALB_RS31375, SALB_RS31405, and SALB_RS31345) encoding PKS were found to be mutated. In addition, a mutation occurred in the diaminopimelate decarboxylase (encoded by SALB_RS25230, *lysA*), which could catalyze the conversion of 2,6-diaminoheptanedioate to l-lysine, the precursor of ε-PL. The mutation of the gene might promote l-lysine production and, consequently, enhance ε-PL production.

### 3.4. Metabolomic Analysis

Non-targeted metabolomic analysis of intracellular metabolites between the original *S. albulus* CICC 11022 and mutant *S. albulus* C214 was carried out. As summarized in Table 2, fourteen metabolites with well-defined functions in central carbon metabolism, l-lysine biosynthesis and degradation, and lipids and relative amino acids were identified. Among these, eleven exhibited upregulation while three showed downregulation.

#### 3.4.1. Central Carbon Metabolism

Compared to the original strain, the mutant exhibited a decreased relative level of glucose 6-phosphate and an increased relative level of gluconic acid, indicating a redirection of carbon skeletons toward the pentose phosphate pathway (PPP). The PPP plays a pivotal role in supplying NADPH and pentoses for cell growth and metabolite biosynthesis [11]. This result was in accordance with the enhanced cell growth observed in the mutant (Figure 2b). The level of succinate was elevated in the mutant, potentially due to the increase in propionyl-CoA conversion to succinate. The elevated succinate levels contribute to intracellular ATP production and oxaloacetate synthesis. Intracellular ATP is crucial for ε-PL synthesis, playing a pivotal role in cellular resistance against auto-acidification stress while facilitating the production of ε-PL [35].

#### 3.4.2. l-Lysine Biosynthesis and Degradation

Oxaloacetate serves as a precursor for the biosynthesis of l-lysine via the 2,6-diaminoheptanedioate pathway (DAP). Moreover, the levels of α-ketoglutarate and N6-(l-1,3-Dicarboxypropyl)-l-lysine decreased, while the levels of l-2-aminoadipate and l-lysine increased. These findings suggest that l-lysine biosynthesis may also occur through the LysW-mediated pathway of l-2-aminoadipate. The l-asparagine could be converted to l-aspartate through the action of asparaginase, leading to the generation of l-lysine via the DAP [8]. As a result, the intracellular levels of l-lysine were found elevated in the mutant, resulting in a corresponding increase in its degradation product, 5-acetamidovalerate.

#### 3.4.3. Lipids and Relative Amino Acids

The metabolomics analysis revealed that four metabolites related to lipid synthesis were significantly upregulated in *S. albulus* C214 compared to *S. albulus* 11022. Myristic acid, a saturated fatty acid, was enriched after ALE. Previous studies have shown that under antibiotic and acid stresses, the ratios of saturated fatty acids in cell membranes increased, thereby enhancing membrane stability [36,37]. The *FASN* was found mutated in *S. albulus* C214 (Section 3.3.3), which could facilitate the synthesis of myristic acid. The upregulation of N-Acetyl-l-citrulline and citrulline could enhance the synthesis of ornithine. Subsequently, ornithine lipids were synthesized by lyso-ornithine lipid O-acyltransferase, an enzyme that was also mutated in *S. albulus* C214 (Section 3.3.3) [28]. Therefore, the upregulation of three metabolites, namely N-Acetyl-l-citrulline, citrulline, and ornithine, along with the mutation of *olsA*, would ultimately enhance the synthesis of ornithine lipids.

## 4. Conclusions

**Figure 3:**
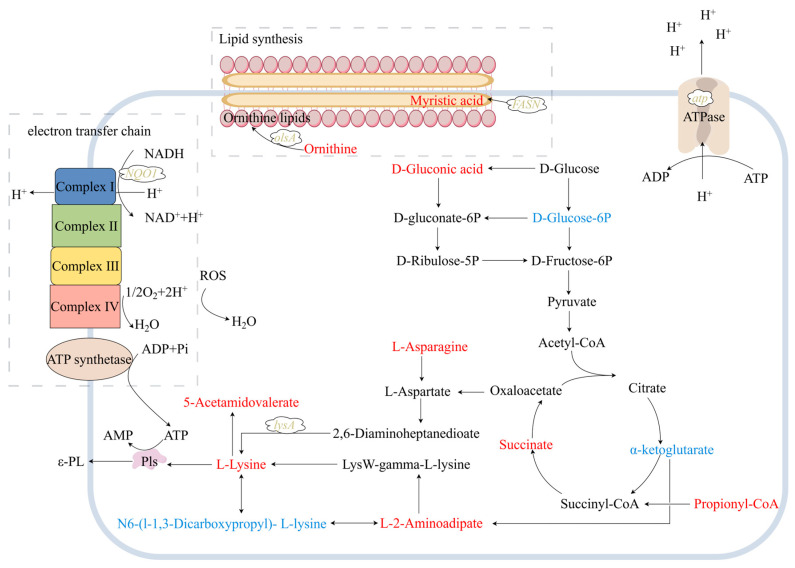
Simplified metabolic network for ε-PL production by *S. albulus*. The metabolites denoted by red represent upregulation, while those denoted by blue represent downregulation. Genetic mutations are represented by yellow.

A mutant strain, *S. albulus* C214, exhibiting enhanced ε-PL production and cell growth, was obtained after ALE using a combination of acidic pH and antibiotics (rifampicin, gentamicin, and streptomycin) as evolution pressures. Comparisons were conducted to reveal the mechanism of high ε-PL production. Both genomic and metabolomic analyses showed many changes. Among them, the accumulation of certain important metabolites and the mutation of *lysA* could enrich the pool of precursor l-lysine, while the mutation of *NQO1* could enhance energy supply, along with the mutation of *atp*, which could create a favorable intracellular pH for the function of ε-PL synthetase. Together, these factors contribute to the elevated production of ε-PL. Moreover, the mutation of *FASN* could improve the synthesis of myristic acid. Additionally, mutations in *olsA* along with elevated levels of ornithine could enhance the production of ornithine lipids. These modifications collectively improve cellular resistance to antibiotics and acid stresses (Figure 3). The information obtained is important for the breeding of ε-PL-producing strains and reveals the pivotal element in ε-PL biosynthesis.

## Figures and Tables

**Table 1:** SNPs and InDel analyses of *S. albulus* C214 and *S. albulus* CICC 11022.

Classification	Gene	Mutation	pos	SNP/Indel		Function
				WT	MT	
Transcriptional regulation	SALB_RS22375	nonsynonymous	4370355	T	C	RNA polymerase sigma-70 factor, ECF subfamily
	SALB_RS03255	nonsynonymous	12915	G	A	XRE family transcriptional regulator
	SALB_RS15530	nonsynonymous	2788378	G	A	MerR family transcriptional regulator
	SALB_RS11990	stoploss	1999574	A	AC	LysR family transcriptional regulator
Transporter	SALB_RS20610	insertion	3943524	C	CG	ABC transporter substrate-binding protein
	SALB_RS11770	insertion	1954790	C	CG	ATP-binding protein
	SALB_RS21360	insertion	4138325	G	GC	MFS transporter
	SALB_RS43140	insertion	1787771	G	GC	MFS transporter
	SALB_RS42470	insertion	2245592	G	GC	MFS transporter
	SALB_RS43175	insertion	3276917	G	GC	MFS transporter
	SALB_RS21805	insertion	4234696	T	TG	MFS transporter
	SALB_RS43140	insertion	1787771	G	GC	MFS transporter, NRE family
	SALB_RS31895	insertion	1049041	G	GC	ATPase
Cell envelope	SALB_RS17270	insertion	3189305	A	AG	lyso-ornithine lipid O-acyltransferase
	SALB_RS01500	insertion	71361	T	TG	[acyl-carrier-protein] S-malonyltransferase
	SALB_RS43310	insertion	21999	C	CG	fatty acid synthase
	SALB_RS28520	insertion	248414	G	GC	cholesterol oxidase
	SALB_RS44660	insertion	3949616	C	CG	CDP-glycerol glycerophosphotransferase
Energy metabolism	SALB_RS44690	nonsynonymous	5057006	A	G	NAD(P)H dehydrogenase
			5057027	A	G	
		insertion	5056987	T	TR	
	SALB_RS44480	nonsynonymous	190230	C	G	AMP-dependent synthetase
		insertion	190228	G	GGC	
	SALB_RS37945	insertion	2444045	G	GC	FAD-binding oxidoreductase
Secondary metabolite synthesis	SALB_RS42445	nonsynonymous	1741660	G	C	polyketide synthase
	SALB_RS31335	insertion	878148	C	CG	polyketide synthase, PKSL
	SALB_RS31375	insertion	909312	G	GC	polyketide synthase. PKSJ
	SALB_RS31405	insertion	927895	G	GC	polyketide synthase, PKSM
	SALB_RS31345	insertion	884938	G	GC	polyketide synthase, PKSM
	SALB_RS25230	insertion	4982823	C	CG	diaminopimelate decarboxylase

WT, *S. albulus* CICC 11022; MT, *S. albulus* C214.

**Table 2:** Effect of ALE mutagenesis treatment on endogenous metabolites determined by LC-MS.

Classification	Metabolites	*m*/*z*	Rt(s)	Formula	Log2 Ratio (MT/WT)
Central carbon metabolism	Glucose 6-phosphate	261.0375	92	C_6_H_13_O_9_P	−1.08 *
	Gluconic acid	197.154	636.5	C_6_H_12_O_7_	2.2 ***
	Succinate	119.0357	291.6	C_4_H_6_O_4_	4.38 **
	Propionyl-CoA	822.1295	330.9	C_24_H_40_N_7_O_17_P_3_S	0.86 *
	α-ketoglutarate	146.0301	71.6	C_5_H_6_O_5_	−1.68 **
l-Lysine biosynthesis and degradation	N6-(l-1,3-Dicarboxypropyl)-l-lysine	277.1392	87.2	C_11_H_20_N_2_O_6_	−0.4 *
	l-Asparagine	133.1023	635	C_4_H_8_N_2_O_3_	3.82 *
	l-2-Aminoadipate	142.0511	102.1	C_6_H_11_NO_4_	0.74 *
	l-Lysine	147.1133	77.6	C_6_H_14_N_2_O_2_	2.55 ***
	5-Acetamidovalerate	159.0922	401.5	C_7_H_13_NO_3_	4.43 **
Lipids and relative amino acids	Myristic acid	229.1797	703.2	C_14_H_28_O_2_	1.99 ***
	N-Acetyl-l-citrulline	217.1181	130.9	C_8_H_15_N_3_O_4_	4.2 *
	Citrulline	176.1032	89.7	C_6_H_13_N_3_O_3_	2.06 *
	Ornithine	133.0975	77.6	C_5_H_12_N_2_O_2_	3.1 ***

*, *p* < 0.05; **, *p* < 0.01; ***, *p* < 0.001. WT, *S. albulus* CICC 11022; MT, *S. albulus* C214.

## Data Availability

The data that support the findings of this study are available from the corresponding author, upon reasonable request.
